# The Clinical and Biochemical Predictors of Bone Mass in Preterm Infants

**DOI:** 10.1371/journal.pone.0165727

**Published:** 2016-11-02

**Authors:** Justyna Czech-Kowalska, Edyta Czekuc-Kryskiewicz, Pawel Pludowski, Katarzyna Zaniuk, Maciej Jaworski, Anna Łuba, Karolina Grzybowska, Krystyna Piłat, Anna Dobrzanska

**Affiliations:** 1 Department of Neonatology and Neonatal Intensive Care, The Children’s Memorial Health Institute, Warsaw, Poland; 2 Department of Biochemistry, Radioimmunology, and Experimental Medicine, The Children’s Memorial Health Institute, Warsaw, Poland; 3 Department of Neonatology, The Holy Family Hospital, Warsaw, Poland; University of Arkansas for Medical Sciences College of Pharmacy, UNITED STATES

## Abstract

**Background:**

Metabolic bone disease of prematurity still occurs in preterm infants, although a significant improvement in neonatal care has been observed in recent decades. Dual-energy X-ray absorptiometry (DXA) is the precise technique for assessing bone mineral content (BMC) in preterm infants, but is not widely available.

**Aim:**

To investigate the clinical and biochemical parameters, including bone metabolism markers as potential predictors of BMC, in preterm infants up to 3 months corrected age (CA).

**Materials and Methods:**

Ca-P homeostasis, iPTH, 25-hydroxyvitamin D, osteocalcin, N-terminal propeptide, cross-linked C-telopeptide and amino-terminal pro C-type natriuretic peptide and the DXA scans were prospectively performed in 184 preterm infants (≤ 34 weeks’ gestation) between term age and 3 mo CA. Lower bone mass was defined as BMC below or equal to respective median value for the whole study group, rounded to the nearest whole number.

**Results:**

The appropriate quality DXA scans were available for 160 infants (87%) examined at term and for 130 (71%) tested at 3 mo CA. Higher iPTH level was the only independent predictor of lower BMC at term, whereas lower BMC at 3 mo CA was associated both with lower urinary phosphate excretion and higher serum osteocalcin level. ROC analysis showed that iPTH >43.6 pg/mL provided 40% sensitivity and 88% specificity in identification of preterm infants with lower BMC at term. In turn, urinary phosphate excretion (TRP>97% or UP/Cr ≤0.74 mg/mg) and serum osteocalcin >172 ng/mL provided 40% sensitivity and 93% specificity in identification of infants with decreased BMC at 3 mo CA.

**Conclusion:**

Serum iPTH might to be a simple predictor of reduced BMC in preterm infants at term age, but urinary phosphate excretion and serum osteocalcin might predict reduced BMC at 3 mo CA. These results represent a promising diagnostic tool based on simple, widely available biochemical measurements for bone mass assessment in preterm infants.

## Introduction

Metabolic bone disease of prematurity (MBD) characterised by low bone mass still occurs in preterm infants, although the significant improvement in neonatal care has been observed in recent decades. The risk of MBD increases with decreasing gestational age and birth weight [1, 2], but the main etiological factor is insufficient Ca and/or P intake [3]. MBD is also more frequent in premature babies on prolonged total parenteral nutrition, on mechanical ventilation due to chronic lung disease, and after furosemide and steroid treatment [4]. However, MBD can also result, at least in part, from insufficient mechanical stimulation for bone formation, due to lower physical activity than in utero. Unfortunately, none of the single biochemical parameters is universally accepted for the diagnosis of MBD in preterm infants. Measurements of serum Ca, P, alkaline phosphatase (ALP), parathormon (PTH), urinary calcium or urinary phosphate excretion are used in clinical practice or are proposed as screening tests of MBD in preterm infants; however, the sensitivity and specificity of those tests are questionable [5–9].

The assessment of biochemical indices of bone formation and resorption is an important methodological advance. Bone formation markers include products of collagen type I synthesis: procollagen type I N-terminal and C-terminal propeptides (PINP and PICP), bone alkaline phosphatase (b-ALP) and non-collagenous protein osteocalcin (OC). OC also plays an important role in energy metabolism as a bone-derived hormone. Bone resorption markers mainly comprise collagen type I degradation products: cross-linked N- and C-telopeptides (NTx and CTx) [10, 11]. Additionally, C-type natriuretic peptide plays an essential role in bone and cartilage growth, while its stable product, amino-terminal propeptide of C-type natriuretic peptide (NT-proCNP), serves as a growth plate activity marker [12]. Apart from biochemical assessment, quantitative bone mass measurement is the most important. Dual-energy X-ray absorptiometry (DXA) is the most accurate, precise and noninvasive technique for assessing bone mass, but the effectiveness of this technique is limited in clinical practice [13, 14].

We hypothesised that the use of a complex algorithm based on clinical characteristic, biochemical markers of Ca-P homeostasis and bone metabolism markers would facilitate the identification of preterm infants at risk of reduced bone mass. This selected group of preterm infants might need special nutritional intervention and might require referral to a specialist centre for bone mass assessment by DXA. The selection of high risk preterm infants using a complex diagnostic approach might be useful in clinical practice.

## Subjects and Methods

### Subjects

The present study was designed as a prospective study of two cohorts of Caucasian preterm infants born <34 weeks of postmenstrual age (PMA) to assessed Ca-P homeostasis, bone metabolism and mineralisation between term age (40 weeks PMA) and 3 months corrected age (CA). A total of 184 preterm infants admitted to the Neonatal Department (The Children’s Memorial Health Institute), Warsaw, Poland) were recruited between August 2004 and February 2008 (historical cohort), and between February 2011 and April 2013. Infants with major congenital abnormalities, chromosomal abnormalities and inborn metabolic disorders were excluded from the study. The study was conducted according to the guidelines laid down in the Declaration of Helsinki, and all procedures involving patients were approved by the Ethics Committee of The Children’s Memorial Health Institute. Written informed consent was obtained from the parents of each participant.

### Methods

Data on delivery characteristics (gestational age, weight and length at birth, multiple gestation) and neonatal characteristics between birth and term age (duration of total parenteral nutrition, type of feeding, time of mechanical ventilation, steroid treatment, diagnosis of bronchopulmonary dysplasia (BPD) were extracted from the infants’ medical records. Clinical examination (craniotabes–soft skull), anthropometric measurements (weight, length, head circumference) biochemical analysis and bone mass measurements, were performed twice at term age (40 weeks postmenstrual age) and at 3 mo corrected age (CA), so 3 mo post-term age. Body weight and body length z- scores at birth and at term age were calculated according to the Fenton Preterm Growth Chart [15].

### Dual-Energy X-Ray Absorptiometry (DXA)

Total body bone mineral content (BMC, g), bone mineral density (BMD, g/cm2) and bone area (BA, cm2) were measured by dual energy X-ray absorptiometry (Lunar Prodigy Advance, GE Healthcare, Madison, United States) at term age and at 3 mo CA. DXA scans were obtained during spontaneous sleep using special infant software provided by the manufacturer. Incomplete DXA scans or with severe movement artefacts were excluded from the statistical analysis.

### Laboratory Measurements

At term age and at 3 mo CA, serum alkaline phosphatase activity (ALP) and calcium (Ca), phosphate (P) and creatinine (Cr) levels were measured in blood and spot urine samples. Urinary calcium to creatinine ratio (UCa/Cr), urinary phosphates to creatinine ratio (UP/Cr) and tubular P reabsorption (TRP = [1-(UP/P)x(Cr/UCr)]x100%) were calculated. Total 25-hydroxyvitamin D level (25OHD) was determined using an immunochemiluminescent method (LIAISON, DiaSorin) with the intra- and inter-assay precision of <8% and <11%, respectively. Intact PTH (iPTH) was assayed using an immunoradiometric kit (IRMA; CIS Bio International, Gif-Sur-Yvette Cedex, France) with inter-assay coefficients of variation (CVs) <7.5%.

For the evaluation of bone formation, serum OC and PINP levels were measured automatically using chemiluminescence immunoassays (Elecsys; Roche Diagnostics, Poland). The intra- and inter-assay CVs for OC were <4.0% and <6.5%, respectively, and for PINP were <2.9% and <3.7%, respectively. For the evaluation of bone resorption, the concentration of serum CTx was assayed automatically using chemiluminescence immunoassay. The intra- and inter-assay CVs were <4.6% and <4.7%, respectively. The serum NT-proCNP level was measured in duplicates using enzyme immunoassay (ELISA, Biomedica, Poland) with inter-assay CV <9.0%.

### Statistics

Lower bone mass was defined as BMC below or equal to respective median value for the whole study group, rounded to the nearest whole number. Intergroup differences of continuous variables were verified with the Student t-test or the Mann-Whitney U-test. The significance of associations between perinatal, clinical or laboratory parameters and lower bone mass was verified by univariate logistic regression analysis. Odds ratios (OR) for the co-occurrence of each explanatory variable and bone mass deficiency were calculated, along with their 95% confidence intervals (95% CI). The variables that showed significant associations with lower bone mass on univariate analysis were subjected to multivariate logistic regression analysis. The variables that showed significant associations with lower bone mass on multivariate analysis were subjected to ROC analysis. In the case of continuous variables, their cut-off values, characterised by the lowest error rate, were determined. The sensitivity and specificity of these cut-off values were calculated, as well as the areas under the ROC curves (AUC) with their 95% CI. Whenever more than one variable showed a significant association with lower BMC on multivariate analysis, the diagnostic accuracy of the composite measure including all these variables was also tested by the ROC analysis. In such cases, the expected values from multivariate logistic regression analysis were assessed. The AUC values for single explanatory variables and their combinations were compared by means of the Z-test. All calculations were carried out with the Statistica 10 package (StatSoft, USA), with the threshold of statistical significance set at p ≤ 0.05. The results are presented as the mean ± SD, unless otherwise indicated.

## Results

A total of 184 preterm infants born below 34 weeks of gestation were included in the study. The final analysis was performed on 160 infants (87%) at term age and 130 infants (71%) at 3 mo corrected (CA) with acceptable DXA scans. The baseline clinical characteristics and biochemical measurements of the study cohort and subgroups comparisons at term age are presented in Table 1.

**Table 1:** Baseline clinical characteristics and biochemical markers of study cohort of preterm infants and a comparison between infants with lower bone mass (BMC ≤ 49 g) and higher bone mass (BMC > 49 g) at term age (40 weeks postmenstrual age). (Data are presented as the mean ± SD or number (%). The statistically significant p value are ≤ 0.05.).

Variable	Total (n = 160)	BMC≤49g (n = 83)	BMC>49g (n = 77)	p–value	Odds ratio (95%CI)*		p- value
**At birth**							
**Gestational age (wks)**	28.4 ± 2.51	28.1 ± 2.43	29.0 ± 2.57	0.015	0.87 (0.76–0.99)	0.030	
**Birth weight (g)**	1139 ± 365	1020 ± 298	1287 ± 382	<0.001	0.09 (0.03–0.29)	<0.001	
**Birth weight z-score**	-0.02 ± 0.86	-0.21 ± 1.00	0.21 ± 0.63	0.008	0.54 (0.36–0.81)	0.003	
**Historical cohort (n)**	115 (62.5%)	51 (61.4%)	43 (55.8%)	0.423	1.34 (0.71–2.53)	0.365	
**Gender: female (n)**	84 (45.7%)	46 (56.6%)	32 (40.3%)	0.060	1.84 (0.98–3.46)	0.058	
**At term age (~40 weeks PMA)**							
**BPD (n)**	86 (46.7%)	43 (53.0%)	28 (35.1%)	0.109	1.97 (1.04–3.73)	0.036	
**Steroids (days)**	1.65 ± 4.10	1.42 ± 3.34	0.91 ± 2.21	0.230	1.07 (0.95–1.21)	0.270	
**Ventilation (days)**	13.2 ± 17.2	14.7 ± 19.2	10.0 ± 13.7	0.092	1.02 (1.00–1.04)	0.090	
**TPN (days)**	21.3 ± 16.8	23.6 ± 17.7	16.1 ± 11.4	0.002	1.04 (1.01–1.07)	0.004	
**Current age (wks)**	11.7 ± 2.94	12.1 ± 3.01	11.4 ± 2.93	0.154	1.01 (1.00–1.03)	0.178	
**PMA (wks)**	40.2 ± 1.92	40.1 ± 1.97	40.4 ± 1.79	0.205	0.93 (0.79–1.10)	0.414	
**Weight (g)**	3005 ± 653	2783 ± 550	3329 ± 566	<0.001	0.09 (0.03–0.29)	<0.001	
**Length (cm)**	49.5 ± 3.76	48.3 ± 3.27	50.8 ± 3.77	<0.001	0.81 (0.73–0.90)	<0.001	
**BMC (g)**	48.2 ± 10.7	40.15 ± 6.51	56.9 ± 6.81	<0.001	N/A	N/A	
**BMD (g/cm2)**	0.23 ± 0.05	0.21 ±0.05	0.25 ± 0.05	<0.001	1.4-7- (1.1-10-1.7–4)	<0.001	
**BA (cm2)**	216 ± 53.2	198 ± 47.2	236 ± 52.6	<0.001	0.02 (0.01–0.13)	<0.001	
**Craniotabes n (%)**	36 (19.6%)	26 (33.3%)	8 (11.0%)	0.001	4.21 (1.74–10.14)	0.001	
**Serum Ca (mmol/L)**	2.40 ± 0.16	2.37 ± 0.16	2.45 ± 0.14	0.001	0.02 (0.00–0.25)	0.002	
**Serum P (mmol/L)**	2.04 ± 0.29	2.01 ± 0.29	2.09 ± 0.25	0.046	0.29 (0.08–1.02)	0.052	
**UCa/Crea (mg/mg)**	0.97 ± 0.87	0.88 ± 0.78	1.15 ± 0.95	0.038	0.69 (0.47–1.01)	0.054	
**UP/Crea (mg/mg)**	1.46 ± 1.40	1.44 ± 1.37	1.30 ± 1.17	0.800	1.09 (0.85–1.40)	0.479	
**TRP (%)**	93.5 ± 7.21	93.5 ±6.46	94.9 ± 5.02	0.438	0.96 (0.91–1.01)	0.142	
**ALP (U/L)**	494 ± 283	560 ± 357	411 ± 122	0.004	4.69 (1.79–12.28)	0.002	
**25-OHD (ng/mL)**	43.0 ± 29.2	44.1 ± 30.6	41.2 ± 22.3	0.983	1.00 (0.99–1.02)	0.496	
**iPTH (pg/mL)**	39.5 ± 46.5	44.3 ± 42.1	24.7 ± 14.8	0.002	1.03 (1.01–1.05)	0.001	
**OC (ng/mL)**	151 ± 61	162 ± 69	146 ± 52	0.210	1.62 (0.71–3.69)	0.246	
**CTx (ng/mL)**	0.93 ± 0.27	0.95 ±0.27	0.90 ± 0.26	0.249	2.09 (0.61–7.15)	0.235	
**PINP (ng/mL)**	5753 ± 1857	5966 ± 1776	5494 ± 1615	0.092	2.49 (0.83–7.43)	0.101	
**NTproCNP (pmol/L)**	64.8 ± 32.7	66.8 ± 37.0	60.7 ± 28.4	0.653	1.01 (0.99–1.02)	0.319	

*The results of univariate logistic regression analysis. BPD–bronchopulmonary dysplasia, TPN- total parenteral nutrition, PMA–postmenstrual age, BMC- bone mineral content, BMD- bone mineral density, BA- bone area, iPTH- intact parathormone, UCa/Cr—urinary calcium to creatinine ratio, UP/Cr—urinary phosphates to creatinine ratio, TRP- tubular reabsorptiom of phoasphates, ALP- serum alkaline phosphatase activity, OC—serum osteocalcin, CTx- serum carboxyterminal cross-linked telopeptide of type 1 collagen, PINP- serum procollagen type 1 N-terminal propeptide, NT-proCNP–serum amino-terminal propeptide of C-type natriuretic peptide, N/A- not applicable

### Predictors of the Lower BMC at Term Age (~40 Weeks PMA)

When using a univariate logistic regression analysis to calculate OR and 95% CI, a significant association between the lower BMC at term gestation and birth weight, intrauterine growth retardation (z-score), gestational age, the diagnosis of bronchopulmonary dysplasia (BPD), the duration of parenteral nutrition, body weight and length at DXA examination, the craniotabes, and the iPTH level, serum Ca and ALP, were observed (Table 1). Craniotabes and ALP had the strongest effect on the infants BMC (Table 1). However, in the multivariate logistic regression analysis only a higher iPTH level remained a significant independent risk factor of the lower BMC in preterm infants at term (OR 1.03, 95% CI 1.00–1.05, p = 0.041).

In the ROC analyses, the iPTH level was the best predictor of the lower BMC (≤ 49 g) at term age. The area under the ROC curve (AUC) was 0.647 (95%CI 0.560–0.734) and the optimal cut-off point for serum iPTH level was 43.6 pg/mL. At the iPTH level > 43.6 pg/mL, a sensitivity of 39.7% and a specificity of 88.2% were obtained.

### Predictors of Lower BMC at 3 Mo Corrected Age (CA)

Children with lower BMC (≤ 91 g) at 3 mo CA had a lower body weight and length, higher urinary excretion (lower UP/Cr and higher TRP), and higher serum OC and PINP levels (Table 2). However in the multivariate logistic regression analysis only lower urinary phosphate excretion (for UP/Cr: OR 0.63, 95%CI 0.01–0.32, p = 0.024; for TRP: OR 1.77, 95%CI 1.15–2.73, p = 0.009) and a higher serum OC level (OR 6.14, 95%CI 1.27–29.77, p = 0.022) remained significant independent predictors of lower BMC in preterm infants at 3 mo CA.

**Table 2:** A comparison of the clinical characteristics and biochemical markers of preterm infants with lower bone mass (BMC ≤ 91 g) and higher bone mass (BMC > 91 g) at 3 months corrected age (CA) and the results of univariate logistic regression analysis. (Data are presented as mean ± SD or number (%).The statistically significant p value are ≤ 0.05.)

Variable	Total (n = 130)	BMC≤91g (n = 64)	BMC>91g (n = 66)	p value	Odds ratio (95%CI)*	p value
**At birth**						
**Gestational age (wks)**	28.7 ± 2.47	28.8 ± 2.58	28.7 ± 2.38	0.879	1.02 (0.89–1.17)	0.782
**Birth weight (g)**	1176 ± 354	1129 ± 317	1223 ± 383	0.209	0.48 (0.16–1.48)	0.198
**Birth weight z-score**	-0.01 ± 0.86	-0.14 ± 1.03	0.11 ± 0.63	0.282	0.71 (0.46–1.07)	0.100
**Historical cohort (n)**	77 (59.2%)	34 (53.1%)	43 (65.2%)	0.212	0.61 (0.30–1.24)	0.164
**Gender: female (n)**	62 (47.7%)	35 (54.7%)	27 (40.9%)	0.160	1.74 (0.86–3.52)	0.117
**At term age (~40 weeks PMA)**						
**BPD (n)**	57 (43.9%)	27 (42.2%)	30 (45.5%)	0.727	0.88 (0.44–1.76)	0.707
**Steroids (days)**	1.70 ± 4.35	2.11 ± 5.39	1.30 ± 3.05	0.274	1.05 (0.96–1.14)	0.303
**Ventilation (days)**	11.8 ± 16.9	10.8 ± 14.4	12.8 ± 19.1	0.623	0.99 (0.97–1.01)	0.505
**TPN (days)**	19.7 ± 15.5	19.7 ± 17.1	19.7 ± 14.0	0.849	1.00 (0.97–1.03)	0.996
**Baseline iPTH (pg/mL)**	33.8 ± 35.2	32.7 ± 34.0	34.9 ± 36.5	0.246	1.00 (0.99–1.01)	0.718
**At 3 mo corrected age**						
**Current age (wks)**	24.2 ± 3.07	24.0 ± 3.34	24.6 ± 2.81	0.307	0.99 (0.98–1.01)	0.308
**PMA (weeks)**	52.9 ±1.50	52.7 ± 1.64	53.2 ± 1.33	0.160	0.82 (0.64–1.05)	0.107
**Weight (g)**	5534 ± 945	5210 ± 722	5849 ± 1031	<0.001	0.02 (0.00–0.19)	0.001
**Length(cm)**	60.1 ± 3.85	58.8 ± 3.53	61.33 ± 3.77	<0.001	0.83 (0.74–0.92)	<0.001
**BMC (g)**	95.3 ± 29.8	73.4 ± 12.5	117 ± 26	<0.001	N/A	N/A
**BMD (g/m2)**	0.38 ± 0.07	0.38 ± 0.04	0.37 ± 0.09	0.787	1.99 (0.01–287)	0.785
**BA (m2)**	269 ± 125	195 ± 30.2	340 ± 139	<0.001	1.21–6 (1.72-9-8.8–4)	p<0.001
**Craniotabes, n (%)**	23 (17.7%)	13 (21%)	10 (15.9%)	0.493	1.44 (0.57–3.61)	0.437
**Serum Ca (mmol/L)**	2.56 ± 0.11	2.55 ± 0.09	2.57 ± 0.13	0.375	0.27 (0.01–7.10)	0.425
**Serum P (mmol/L)**	2.04 ± 0.20	2.01 ± 0.20	2.08 ± 0.20	0.136	0.18 (0.03–1.18)	0.070
**UCa/Cr (mg/mg)**	0.49 ± 0.42	0.49 ± 0.45	0.45 ± 0.41	0.728	1.07 (0.47–2.46)	0.863
**UP/Cr (mg/mg)**	1.09 ± 1.12	0.81 ± 0.86	1.37 ± 1.28	<0.001	0.55 (0.35–0.86)	0.009
**TRP (%)**	95.6 ± 5.32	97.0 ± 2.97	94.3 ± 6.63	<0.001	1.21 (1.07–1.38)	0.002
**ALP (U/L)**	423 ±122	426 ± 127	419 ± 118	0.838	1.12 (0.33–3.86)	0.853
**25-OHD (ng/mL)**	45.4 ± 25.6	48.9 ± 32.7	41.8 ± 15.0	0.950	1.01 (1.00–1.03)	0.123
**PTH (pg/mL)**	22.6 ±23.0	24.6 ± 27.3	20.5 ± 17.4	0.426	1.01 (0.99–1.03)	0.348
**OC (ng/mL)**	140 ± 54.2	158 ± 64.2	120 ± 32.0	0.001	9.72 (2.54–37.18)	0.001
**CTx (ng/mL)**	0.82 ± 0.21	0.85 ± 0.22	0.79 ± 0.18	0.081	4.06 (0.59–27.73)	0.149
**PINP (ng/mL)**	3143 ± 870	3310 ± 942	2973 ± 760	0.047	3.69 (0.88–15.52)	0.071
**NT-proCNP (pmol/L)**	42.9 ± 18.0	43.2 ± 16.4	42.49 ± 19.6	0.672	1.00 (0.98–1.03)	0.843

*The results of univariate logistic regression analysis. BPD–bronchopulmonary dysplasia, TPN- total parenteral nutrition, PMA–postmenstrual age, BMC- bone mineral content, BMD- bone mineral density, BA- bone area, iPTH- serum intact parathormone, UCa/Cr—urinary calcium to creatinine ratio in the spot urine sample, UP/Cr—urinary phosphate to creatinine ratio in the spot urine sample, TRP- tubular reabsorption of phosphate, ALP- serum alkaline phosphatase activity, OC—serum osteocalcin, CTx- serum carboxyterminal cross-linked telopeptide of type 1 collagen, PINP- serum procollagen type 1 N-terminal propeptide, NT-proCNP–serum amino-terminal propeptide of C-type natriuretic peptide, N/A- not applicable

**Fig 1:**
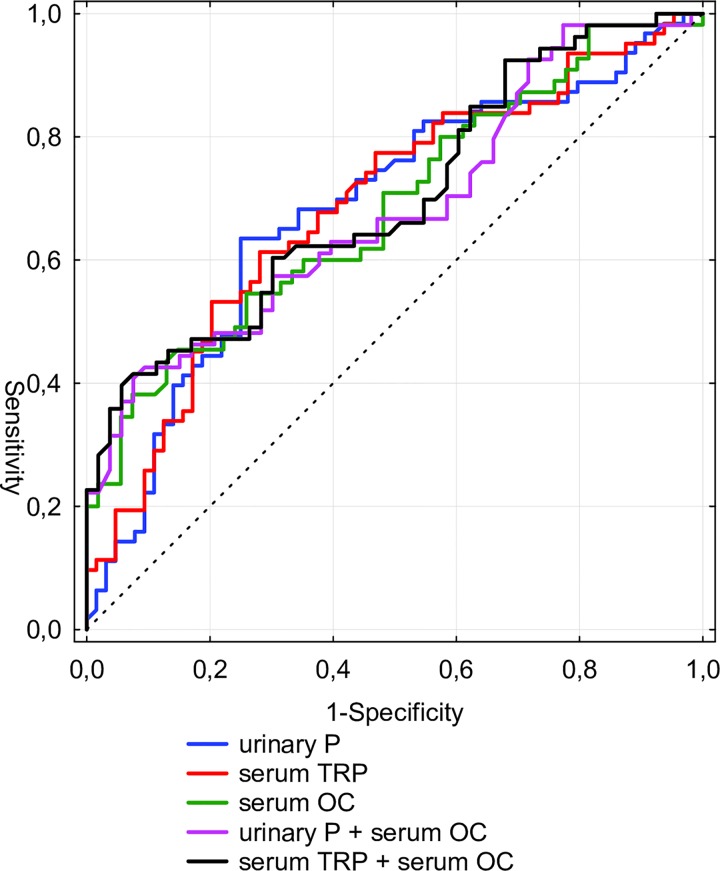
ROC curves for predicting reduced BMC in preterm infants at 3 mo CA. UP/Cr ratio–urinary phosphate to creatinine ratio in the spot urine sample, TRP–tubular reabsorption of phosphate, OC–serum osteocalcin.

In the ROC analyses, UP/Cr ratio, TRP, OC level and the composite algorithms had an around 70% predictive value of the reduced BMC at 3 mo CA (Table 3). There were no significant differences between the AUC of single and composite algorithms in the Z-test (data not presented) (Fig 1.)

**Table 3:** The predictors of lower bone mass (BMC ≤91 g) in 130 preterm infants at 3 corrected age in the ROC analysis.

Parameter*	Cut-off	Sensitivity	Specificity	AUC	(-)95%CI	(+)95%CI
**UP/Cr (mg/mg)**	≤0.74	0.635	0.750	0.682	0.588	0.776
**TRP (%)**	>97	0.613	0.719	0.687	0.594	0.780
**OC (pg/dL)**	>172	0.382	0.926	0.685	0.586	0.784
**UP/Cr (mg/mg) and OC (pg/dL)**	UP/Cr ≤0.74, OC >172	0.407	0.925	0.682	0.581	0.782
**TRP (%) and OC (pg/dL)**	TRP >97; OC >172	0.396	0.943	0.660	0.600	0.789

*UP/Cr—urinary phosphates to creatinine ratio in the spot urine sample, TRP- tubular reabsorptiom of phosphates, OC—serum osteocalcin, AUC- area under the ROC curve

## Discussion

The present prospective cohort study of preterm infants born at < 34 weeks gestation investigated the clinical and biochemical parameters, including bone metabolism markers, as the potential predictors of BMC in preterm infants up to 3 months corrected age. The clinical risk factors of the reduced BMC are commonly encountered in a pathogenesis of MBD. In our study we again confirmed that low gestational age and low birth weight increased the risk of BMD. Indisputably, the frequency of BMD is inversely related to gestational age and birth weight [4, 13, 16, 17]. The long-term consequences of birth weight on BMC should be also underlined. A positive association between birth weight and bone mass was clear among the children, but was unclear among adolescents and weak among adults [18, 19]. Infants born small for their gestational age are also at a higher risk of MBD, and reduced BMC which might persist up to 6 mo CA or even longer [17, 20]. However, in our study the impact of intrauterine growth retardation on BMC is seen only at term—not at 3 mo CA. This difference might be related to the lower percentage of small-for-gestational-age infants in our study. In agreement with other studies we confirmed that total parenteral nutrition is a risk factor in reduced BMC [4, 17, 21]. By contrast, there are conflicting results about the possible influence of the diagnosis of bronchopulmonary dysplasia (BPD) and postnatal steroid treatment on bone mass in preterm infants. Our data suggests that the diagnosis of BPD increases the risk of reduced BMC at term age by 1.97 (95%CI: 1.04–3.73). An association between BPD and MBD was also reported in populations with a high BPD rate but not in populations with a low BPD rate [4, 17, 22]. The diagnosis of BPD might be associated with steroids and diuretic treatment and a longer duration of mechanical ventilation, which are also reported as MBD risk factors [4, 17]. We found no effect of mechanical ventilation and steroids on BMC in our study; nevertheless, short steroid courses were preferred during the study period. Additionally, we found that craniotabes increased the risk of lower BMC by 4 times; however, it was present in only 34% of the infants with lower BMC. Craniotabes might be a clinical sign of bone demineralisation due to calcium, phosphate or vitamin D deficiency, but of limited diagnostic accuracy [23, 24].

There is a lack of one precise biochemical marker of MBD in preterm infants available in clinical practice [5]. Despite that, the diagnosis of MBD is based almost universally in the US on an elevated ALP [7], although there is no general cut-off ALP level [6, 7, 17, 25]. We should bear in mind that an ALP level of > 600 IU/L is very common in extremely low birth weight infants, therefore the serum ALP might not be a suitable diagnostic tool for such an immature group of preterm infants [16]. Additionally, Faerk et al. did not find any correlation between ALP and BMC assessed by DXA at term gestation [26]. In our study a higher ALP level increases the risk of lower BMC by 4 times at term age, but was not a significant predictor of lower BMC in the multivariate logistic regression analysis or the ROC analysis. In contrast to ALP, the iPTH level so far was not widely validated as a biochemical marker of MBD in preterm infants. In our study, the iPTH level of > 43.6 pg/dL appeared as a predictor of lower BMC at term age, but not at 3 mo CA. Interestingly, van de Lagemmat et al. found a negative association between iPTH and BMC that persisted up to 6 mo CA, although all infants had a normal iPTH level [27]. On the other hand, 82% of extremely low birth weight infants with confirmed MBD by X-ray examination had an elevated iPTH level [28]. It should be underlined that an X- ray examination is relatively insensitive and bone mass might be decreased [5] by at least 30–40% at the time of diagnosis of MBD, while DXA seems to be the most accurate and precise technique for assessing bone mineralisation in preterm infants [13, 29, 30]. It is more likely that an elevated iPTH level in our patients resulted rather from insufficient calcium intake/absorption than their vitamin D status, insofar as we found less serum calcium in the lower BMC group, while there were no differences in the serum 25OHD level between the groups. A decreasing iPTH level during oral calcium supplementation means a higher probability of calcium deficiency in preterm infants with an elevated iPTH [28, 31]. On the other hand, a higher vitamin D intake in preterm infants fed mineral-enriched postdischarge formula is associated with increased bone accretion. Nutritional intervention (increased Ca and vitamin D intake) can suppress PTH synthesis and bone resorption and can improve bone mineralisation. Therefore, an early identification of patients with elevated iPTH could improve the outcome. An iPTH level assessment during hospitalisation might to be an easy and inexpensive diagnostic tool for preterm infants with suspected MBD. However, the question arises of why the iPTH level is not a more important predictor of reduced BMC at 3 mo CA. A decreasing iPTH level between term age and 3 mo CA might be caused by decreasing needs for calcium and a better proportion between dietary supply and demand when preterm infants reach term age.

In the light of the limitation of single predictors of MBD, our approach to the diagnosis of MBD based on a combination of several factors, including bone formation and resorption markers, seems to be more encouraging. Bone formation (OC, PINP) and resorption indices (CTx), as well as a growth plate activity marker (NT-proCNP), were higher in the group of preterm infants with lower BMC both at term (BMC ≤ 49 g) and 3 mo after (BMC ≤ 91 g); however, only OC and PINP reached statistical significance. Increased bone turnover was previously reported in preterm infants, especially those with MBD [32–34]. The higher bone metabolism in the lower BMC group might be compensatory. The reduced BMC group, at baseline as well as at 3 mo CA, was characterised by longer steroid usage. Glucocorticoid treatment is associated with higher bone turnover in preterm infants [35, 36]. The concentrations of bone metabolism markers were independent at the 25OHD level, which was similar between groups. OC alone or together with UP/Cr and TRP seem to be very specific predictors of reduced bone mass in preterm infants at 3 mo CA. OC is a particular marker of bone formation. Serum OC level represents osteoblast metabolic activity and mineral deposition, but is also derived from bone during osteoclast activity. Therefore, OC can serve rather as a marker of bone turnover [37]. An increasing amount of data indicates that OC is an endocrine hormone which regulates energy metabolism, male fertility and brain development [38]. Our data points to OC’s being the best predictor of MBD from evaluated markers of bone metabolism. This phenomenon is independent of vitamin D status (a lack of correlation between OC and 25OHD at 3 mo CA; data not shown). Concentrations of OC might be related to skeletal size and body weight [39, 40]. We noted a correlation between OC and body length at 3 mo CA (but not body weight); however, this correlation was weak and negative (R = -0.224, p = 0.016; data not shown). This correlation corresponded with an observed OC level decrease and body length increase during the study period. Nevertheless, serum OC level, CTx, PINP do not correlate with BMD in children with clinical bone fragility [41, 42]. Similarly, Litmanovitz et al. found that changes in the biochemical markers could not predict the changes in bone strength assessed by quantitative ultrasound [43]. We should bear in mind that MBD might be a mixture of increased bone resorption and decreased bone formation, together with insufficient bone matrix mineralisation. DXA is generally accepted as an accurate and precise non-invasive technique for assessing bone mineralisation [13], whereas new bone formation might not be necessarily followed by bone mineralisation in cases of insufficient supply. Nutritional intervention improved BMC, while a physical activity programme might improve not only BMC but also bone formation, and might reduce bone resorption [13, 44–48].

Unfortunately, the problem of MBD still exists despite improvements in neonatal care. From the clinical point of view the major concern is the lack of a sensitive, widely accessible biochemical marker of MBD. We tried to build a precise diagnostic tool for MBD taking into account the clinical and biochemical measurements. The strength of the study is the wide range of the analysed biochemical markers including calcium, phosphate, ALP, iPTH, 25OHD and bone metabolism markers such as PINP, NTproCNP, CTx and OC. Bone metabolism markers were measured automatically in serum by modern tests with a higher precision than was reported for manual measurements, especially in urine samples [40]. An additional important strength of the study is use of the most precise tool of bone mass assessment (DXA) in preterm infants. However, this study also had some limitations. First, we did not assess all possible clinical determinants of bone mass in preterm infants, including diet due to diversity in enteral nutrition during hospitalisation. Additionally we did not analyse Ca and P intake from parenteral nutrition, which can have an influence on BMC. Furthermore, we did not assess biochemical parameters before 40 weeks PMA. Finally, an extrapolation of our results into the overall population might be biased by the study population recruited in a tertiary neonatal care unit with no maternity unit (only external deliveries).

In conclusion, the serum iPTH might to be a predictor of reduced BMC at term age in preterm infants, whereas urinary phosphate excretion (UP/Cr or TRP) and serum OC might predict lower BMC at 3 mo CA. In our view these results represent a promising diagnostic tool based on simple, widely available biochemical measurements for bone mass assessment in preterm infants.
